# Determinants of Preschool Teachers’ Knowledge-Sharing Behavior from a Thinking Style Perspective

**DOI:** 10.3390/bs13030230

**Published:** 2023-03-06

**Authors:** Lu Cheng, Dang Wu, Junwei Cao

**Affiliations:** 1 Department of Child and Family Studies, Kyungpook National University, Daegu 41566, Republic of Korea; 2 School of Special Education, Handan University, Handan 056000, China; 3 School of Business, Yangzhou University, Yangzhou 225127, China

**Keywords:** preschool teachers, knowledge-sharing behavior, Norm Activation Model, thinking style

## Abstract

Knowledge sharing not only promotes communication among teachers to achieve self-professional growth but also facilitates knowledge innovation. Thus, knowledge sharing among preschool teachers deserves attention. This study explored the factors influencing preschool teachers’ knowledge-sharing behaviors. A questionnaire was administered to 297 preschool teachers using a Norm Activation Model from a thinking style perspective. Data analysis was performed using partial least square-structural equation modelling (PLS-SEM). The findings indicate that executive thinking style preschool teachers showed a significant positive influence of awareness of consequences; legislative thinking style preschool teachers showed a significant positive influence of awareness of consequences and ascription of responsibility; awareness of consequences had a significant positive influence on ascription of responsibility; awareness of consequences and ascription of responsibility had a significant positive influence on personal norms; and personal norms had a significant positive influence on knowledge-sharing behavior. Meanwhile, the influence of executive thinking style on ascription of responsibility, legislative thinking style on ascription of responsibility, and awareness of consequences on personal norms emerged as significantly different among preschool teachers in two different contexts: interpersonal sharing and Internet sharing. This study confirmed the factors influencing preschool teachers’ knowledge-sharing behaviors from a thinking style perspective and provides suggestions for improving preschool teachers’ knowledge-sharing behaviors.

## 1. Introduction

Knowledge sharing has received widespread attention in various fields, including education [1]. This is because knowledge sharing not only serves as a mechanism for interaction and transfer among individuals as well as within groups [2] but also plays a very important role in teacher professional development and educational reform [3].

From the perspective of teachers themselves, as innovators and transmitters of knowledge, they must ensure that their knowledge resources are competitive and valuable through continuous learning [4]. Knowledge sharing allows teachers to gain access to their colleagues’ experiences [5] and generate new ideas [6]. Teachers can help them understand relevant knowledge when answering or discussing colleagues’ queries [7]. Sharers of knowledge benefit more than receivers [8]. From an organizational perspective, knowledge sharing among teachers can improve the competitiveness of school organizations and is an important method of knowledge management in schools [9]. It is also key to improving instructional practice, student learning outcomes, and school systems [10,11].

With the development of information technology [12], new digital resource environments, such as online learning, have gradually formed and matured [13]. Internet knowledge sharing has been more effective in helping teachers solve various problems [7] and in improving their teaching and research skills [4]. Sharing knowledge related to teaching and learning and providing digital resources to students are strategies to better deal with the increasing diversity of students, thus offering potential new approaches to teaching and learning [14]. Therefore, digital resources, such as multimedia courseware, electronic teaching plans, and multimedia materials used by preschool teachers in their teaching practices, have become the main source of shared knowledge.

However, technology alone does not guarantee that teachers engage in knowledge-sharing activities [15]. Some studies have found that the public nature of knowledge in virtual academic communities tends to make sharing unbalanced, and people are more inclined to access free knowledge resources than to share their own knowledge [16]. The personal factor is the most important factor influencing teachers’ knowledge sharing [17]. Therefore, this study analyzes the factors that influence teachers’ knowledge-sharing behavior from an individual perspective.

Researchers have found that fear of criticism hinders teachers’ knowledge-sharing behavior [18]. Studies have found that low psychological safety and psychological empowerment can cause teachers to suppress their own views and refuse to share knowledge [16]. Conversely, factors such as teachers’ self-efficacy [19], personal motivation [12], and attitudes toward knowledge sharing promote knowledge sharing [20]. In addition, thinking style is closely related to self-efficacy [21], motivation [22], and attitude [23]. For example, preservice teachers with a critical-creative thinking style have an impact on self-efficacy [21]. Thus, thinking style could be an antecedent of teachers’ knowledge-sharing behaviors. However, there is a lack of empirical research on the influence of thinking styles on teachers’ knowledge-sharing behaviors, and most of the research on teachers’ knowledge sharing has focused on university teachers, with a lack of empirical research on preschool teachers’ knowledge-sharing behaviors. Therefore, this study analyzes the influence of preschool teachers’ thinking styles on knowledge-sharing behaviors by asking the first research question.

RQ1: How do preschool teachers’ thinking styles influence knowledge-sharing behaviors?

Moreover, Van Acker, Vermeulen, Kreijns, Lutgerink, and Van Buuren [24] divided the contexts in which teachers share knowledge into two: sharing with colleagues at their school (interpersonal sharing) and sharing with the public through the Internet (Internet sharing). The two sharing contexts yielded different results. For example, Van Acker, Vermeulen, Kreijns, Lutgerink, and Van Buuren [24] showed that knowledge is shared twice as often between individuals as it is shared through websites. Wang, Tigelaar, and Admiraal [14] also confirmed the differences between teachers in both within- and outside-school knowledge-sharing contexts. Therefore, this study will distinguish between two sharing contexts: preschool teachers’ sharing with colleagues at their school (interpersonal sharing) and sharing with the public through the Internet (Internet sharing), setting the second research question:

RQ2: What are the differences in the results for preschool teachers in different contexts (interpersonal sharing and Internet sharing)?

This study builds a research model based on the Norm Activation Model and conducts a questionnaire survey and analysis with preschool teachers as research subjects to verify the hypotheses proposed in this study. The potential research contributions of this study are as follows: (1) This study explores the personal factors that influence preschool teachers’ knowledge-sharing behavior. (2) This study verifies the Norm Activation Model through preschool teachers’ knowledge sharing behaviors to support and broaden the applicability of the Norm Activation Model. (3) This study provides empirical evidence of preschool teachers’ knowledge-sharing behavior and reference materials for preschool teachers’ and administrators’ knowledge management.

### 1.1. Theoretical Background and Hypothesis Development

#### 1.1.1. The Norm Activation Model

Pro-social behavior is altruistic and involves helping, sharing, and cooperating [25]. Knowledge-sharing behavior can be considered a pro-social behavior [26] because the motivation for sharing is pro-social [27], and knowledge-sharing is an act of helping others [28]. This pro-social behavior is beyond the scope of one’s own work and is voluntary in nature [29]. Previous studies have used the Theory of Planned Behavior to predict knowledge-sharing behavior [30] in pro-social behavior. However, TPB is mainly concerned with attitude, subjective norm, and perceived behavioral control [31]. Motives of a selfless, altruistic or pro-social nature are not considered, ignoring the presence/activation of personal moral norms [32].

The role of moral obligations is attended to in the Norm Activation Model [33]. NAM is used to predict pro-social behavior [34] and has been widely used in the analysis of various pro-social behaviors. Examples include revisiting volunteer tourists [35]; reducing food waste [36]; adopting electric vehicles [37]; and purchasing energy-saving appliances [38], all employing normative activation models. Therefore, the NAM could be more applicable to the study of preschool teachers’ knowledge-sharing behavior.

According to NAM [34], pro-social behavior comprises three important factors: awareness of consequences, ascription of responsibility, and personal norms. Awareness of consequences refers to whether someone is aware of the negative consequences for others or for other things he values when he does not act pro-socially [25]. Ascription of responsibility is a sense of responsibility for the negative consequences of unsocial behavior [25]. Personal norms refer to ethical obligations to perform or refrain from specific actions [33]. Norm activation theory suggests that personal norms are activated by awareness of the consequences of a person’s actions and the ascription of responsibility for them [39]. In this study, awareness of consequences in preschool teachers’ knowledge-sharing behaviors refers to whether the preschool teacher is aware of the negative consequences of not sharing knowledge with others or to other things he values. Ascription of responsibility refers to preschool teachers’ sense of responsibility for the negative consequences of the act of not sharing knowledge. Personal norms refer to the ethical obligations of preschool teachers to perform or not to perform knowledge-sharing behaviors. Personal norms for preschool teachers’ knowledge-sharing behaviors are activated through awareness of the consequences of not sharing knowledge and the ascription of responsibility to them.

Awareness of consequences as a positive predictor of personal norms has been repeatedly demonstrated in previous studies [36,37]. In this study, the negative consequences of not sharing knowledge among preschool teachers may be detrimental to knowledge innovation [40], alienation from colleagues [10], and lack of access to the latest information [5]. Therefore, if preschool teachers are aware of these negative consequences, this will stimulate the norms of personal knowledge sharing among preschool teachers.

Awareness of consequences positively influences the ascription of responsibility [41]. This means that if preschool teachers do not understand the negative consequences of not sharing knowledge, it will be difficult to form an ascription of responsibility. Conversely, preschool teachers are more likely to develop ascription of responsibility if they are aware of the possible negative consequences of not sharing their knowledge. In addition, ascription of responsibility is a positive predictor of personal norms [36,37]. Preschool teachers’ moral obligation to share knowledge is also triggered when they realize that they are jointly and severally responsible for the adverse consequences of not doing so. Therefore, we hypothesize as follows:

**H1a.**  *Preschool teachers’ awareness of consequences has a positive influence on personal norms*.

**H1b.**  *Preschool teachers’ awareness of consequences has a positive influence on ascription of responsibility*.

**H1c.**  *Preschool teachers’ ascription of responsibility has a positive influence on personal norms*.

Personal norms symbolize a certain moral obligation [33] related to “how one should behave” [42]. Research has confirmed that personal norms positively influence pro-social behavior [35]. Sharing behavior is a form of pro-social behavior [42], and teachers’ knowledge-sharing behavior is not driven by self-interest, is public-spirited, and is driven by their own moral obligations [43,44]. This means that the higher the personal norms of preschool teachers, the higher the likelihood of the knowledge-sharing behavior of preschool teachers. Therefore, we posited the following hypothesis:

**H2.**  *Preschool teachers’ personal norms have a positive influence on knowledge-sharing behavior*.

#### 1.1.2. Thinking Style

Although the NAM was proposed in the context of pro-social behavior, the model predicts pro-social behavior only through awareness of consequences, ascription of responsibility, and personal norms [34], and does not consider the mental mechanisms behind it, such as thinking styles [45]. This study added a thinking style to the Norm Activation Model to compensate for this limitation in order to better explore the determinants of preschool teachers’ knowledge sharing behaviors.

Teachers’ behavioral intentions and thinking styles are closely related [46], and thinking styles have an important influence on teachers’ access to and dissemination of information [47]. Thinking style does not refer to an individual’s abilities or motivations but rather to the characteristics of an individual’s tendency to use his/her abilities [48]. Thinking styles play a very important role in teacher professional development [49] and have been widely used in teacher research. For example, thinking style is related to the attitude of the teaching profession [50]. Teachers’ thinking styles can influence changes in their behavior [51]. Thinking styles can guide teachers’ actions because they can influence cognitive processes, beliefs, and organizational resources, among other ways, to prioritize what they must or should not deal with [52].

Sternberg [45] described how individuals manage or organize their thoughts in terms of mental self-government. Each person has their own mental agency for coping with the thinking process, which is divided into judicial, executive, and legislative. Judicial thinking style is the tendency to make judgments about things or people and to critically assess what is happening. Executive thinking style is the tendency to work on what is assigned to him or to prefer to deal with pre-determined problems. Legislative thinking refers to the tendency to perform tasks in one’s own way [45].

People with a judicial thinking style will study the pros and cons of different views and ideas before putting them into practice [45]. In other words, people with a judicial thinking style are more analytical and critical [45], are able to reflect more on issues, are more rational, and have a lower probability of making mistakes [53]. This means that they will be more cautious and aware of the consequences [54]. Thus, preschool teachers with judicial thinking weigh the possible outcomes of different options and anticipate the potential consequences before deciding whether to share knowledge.

The critical nature of judicial thinking enables people to make their own contributions to society [55]. The attributes of the teaching profession dictate that it is the responsibility of teachers to disseminate knowledge. Knowledge sharing, as one of the ways to disseminate knowledge, is an act by which teachers intentionally disseminate knowledge through critical thinking, interpretation, clarification, and reflection [56]. It can be surmised that preschool teachers with a judicial thinking approach realize that knowledge sharing is the responsibility of being a teacher through judgment and evaluation and have a sense of responsibility for the adverse consequences of not sharing knowledge. Therefore, our hypotheses are as follows:

**H3a.**  *Preschool teachers’ judicial thinking style has a positive influence on awareness of consequences*.

**H3b.**  *Preschool teachers’ judicial thinking style has a positive influence on ascription of responsibility*.

Unlike critical judicial thinking, executive thinking is more inclined to be told what to do [47]; people with executive thinking are more often than not executors, preferring to solve problems with existing solutions [45]. This suggests that people with executive thinking are more likely to be influenced by popular opinion [54] and tend to use socially established rules to deal with problems [57]. The intensity of individual sharing is influenced by collectivist values [27], and individuals are often motivated by collectivism to engage in knowledge-sharing behaviors [58]. Preschool teachers with executive thinking are influenced by the views of the teacher community when the act of knowledge sharing is generally perceived by the preschool teacher community as a way to communicate learning knowledge and they are encouraged to do so. When they see that most preschool teachers engage in knowledge-sharing activities, they may imitate them because they realize that there may be negative consequences for not doing so.

An executive thinking style is usually associated with a conservative style [59], showing a tendency to perform duties in accordance with rules and procedures [60] and do the best possible job [61]. This means that people with executive thinking are clearer about their duties or responsibilities when performing tasks and are therefore better able to complete them. For example, students with executive thinking are more likely to perform well on tests [62]. In addition, the majority of teachers’ thinking styles are executive [63]. Therefore, it can be inferred that preschool teachers with executive thinking are aware that it is their duty to disseminate knowledge and see knowledge sharing as their responsibility. Therefore, we hypothesize as follows:

**H4a.**  *Preschool teachers’ executive thinking style has a positive influence on awareness of consequences*.

**H4b.**  *Preschool teachers’ executive thinking style has a positive influence on ascription of responsibility*.

Legislative thinking can usually be understood as free-thinking [59]. Legislative thinkers do not like to perform tasks in the traditional way and prefer to perform them in a unique, novel, and creative way [60]. Teachers who engage in legislative thinking will create a learning atmosphere that encourages students to discuss different points of view [51]. In addition, legislative thinking teachers possess analytical and reflective thinking in their teaching practice [51]. Thus, legislative thinking preschool teachers will analyze the possible negative effects of not sharing knowledge before acting, and will be more sensitive to the consequences that may arise.

Creative teachers take responsibility for their work practices and beliefs [64], and treat the dissemination of knowledge as their responsibility [56]. Research has shown that teachers with a legislative thinking style can predict classroom teaching effectiveness [65]. This means that teachers with legislative thinking are aware of their responsibilities as teachers and are accountable for their work. Moreover, the nature of the teaching profession requires teachers to disseminate and impart knowledge, and to continually create new knowledge [56]. Knowledge sharing is one way of realizing the nature of the teaching profession, and because of the nature of the teaching profession, preschool teachers who have legislative thinking will consider knowledge sharing their responsibility. Thus, we can hypothesize the following:

**H5a.**  *Preschool teachers’ legislative thinking style has a positive influence on awareness of consequences*.

**H5b.**  *Preschool teachers’ legislative thinking style has a positive influence on ascription of responsibility*.

#### 1.1.3. Sharing Context

Teacher knowledge sharing contexts can be divided into sharing with colleagues at their school (interpersonal sharing) and sharing with the public through the Internet (Internet sharing) [24]. The results may vary across different sharing contexts. Van Acker et al. [24] found that teachers preferred sharing among school colleagues, and that teachers’ intentions to share and trust were higher in school contexts than in Internet contexts. Fear of criticism limits knowledge sharing among teachers in virtual communities [18], and the incentive of egoism makes people more inclined to share knowledge in schools [58]. However, some studies have also suggested that teachers tend to use the Internet to share their knowledge [66]. Therefore, we hypothesize the following:

**H6.**  *Preschool teachers have significantly different results in different contexts (interpersonal sharing and Internet sharing)*.

### 1.2. Research Model

**Figure 1:**
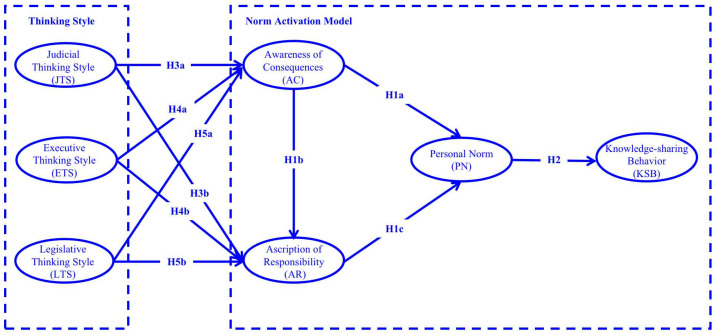
Research model.

This study introduces three thinking styles (judicial, executive, and legislative) based on the original Norm Activation Model, and proposes a model for this study (Figure 1). This study hypothesized that preschool teachers’ thinking styles activate personal norms through awareness of the consequences and ascription of responsibility, which leads to knowledge-sharing behaviors.

## 2. Empirical Analysis

### 2.1. Questionnaire Survey Design

The thinking style scale of Groza, Locander, and Howlett [47] was used directly in this study. The scales of the Norms Activation Model were originally used in various studies of pro-social or pro-environmental behaviors, such as waste separation [41] and electricity saving behavior [67], which are unified in this study as digital resource sharing behavior. Knowledge sharing in the original knowledge sharing scale [68,69] was unified as digital resource sharing. To make the questionnaire more appropriate for this study, we unified the five-point Likert scale from 1 (strongly disagree) to 5 (strongly agree). In addition, we attached great importance to the validity and reliability of the questionnaire. Various methods such as pre-survey, Cronbach’s alpha coefficient, and factor analysis were used to ensure the validity and reliability of the questionnaire during the research design and data analysis. The results of our data analysis showed that the questionnaire had good reliability and validity and could be used to explore our research questions.

To ensure the accuracy of the language in the questionnaire, we invited graduate students in English to edit the language of the questionnaire. In addition, 50 preschool teachers were invited to conduct a pre-survey. Respondents were required to answer all the questions to successfully submit the questionnaire. After completing the questionnaire, the pre-surveyors gave feedback to the researcher on the parts of the questionnaire that were difficult to understand, or their own suggestions, and the researcher adjusted the questionnaire again based on the feedback. The final version of the questionnaire is shown in Appendix A.

The questionnaire was administered in Taiyuan, a city which values digital education. To improve teachers’ digital teaching literacy, Xiaodian District, Taiyuan City, conducted an 11-day training program for 15,000 teachers in preschools, elementary schools, and secondary schools to improve their information skills [70]. We first joined several large local chat groups for preschool teachers through WeChat and Tencent instant messenger. Between 20 December 2022 and 11 January 2023, 100 preschool teachers were randomly recruited through chat groups and asked to forward the questionnaire to five colleagues. The study data were collected anonymously, and the teachers were informed of the purpose and use of this study before filling out the questionnaire. We promised that the questionnaire would not be used for other purposes and received their consent. Teachers received 8 CNY as a reward after completing the questionnaire. In this study, the question of whether they would engage in knowledge-sharing behavior was set on the first page of the questionnaire, and if teachers filled in no, they skipped directly to the last page to end the survey, and these questionnaires were excluded. In addition, we found in the pre-survey that it took at least 2 min and 30 s to fill out the questionnaire completely and carefully, so we excluded questionnaires that took less than 2 min and 30 s and questionnaires that all selected the same option. Therefore, of the 418 questionnaires were returned, and 297 questionnaires were finally analyzed in this study.

### 2.2. Research Methods

The sample of this study was relatively small, and it was an exploratory analysis with seven variables, so the analysis method chosen was partial least squares equation modeling (PLS-SEM). This is because PLS-SEM is suitable for analyzing small samples with more than six variables and is convenient for dealing with non-normally distributed data [71]. We measured and obtained the data distribution using multivariate normality analysis with a web calculator (https://webpower.psychstat.org/, accessed on 1 February 2023). The results were as follows: Mardia’s multivariate skewness was β = 144.261 (*p* < 0.001) and multivariate kurtosis was β = 875.515 (*p* < 0.001), which suggests multivariate non-normality [72]. Therefore, this study was considered appropriate for PLS-SEM as a data analysis method [73].

This study ensures the validity of the data through non-response bias, common method bias, composite reliability (CR), average variance extracted (AVE), discriminant validity, outer loading, and collinearity. The structural model was then tested by examining the significance of the relationship between the variables to verify the hypotheses proposed in this study. In the structural model, *p* < 0.05 indicates that the results are significant and support the hypothesis. Additionally, βs > 0 represents positive influence and βs < 0 represents negative influence. Finally, goodness of fit of the model was ensured by standardized root mean square residuals (SRMR).

## 3. Results

### 3.1. Demographics

From the 297 valid questionnaires collected, 274 (92.3%) of the respondents were female and 23 (7.7%) were male. Of these, the largest proportion was between 26 and 35 years old (*n* = 158, 53.2%), followed by 25 years old and below (*n* = 64, 21.5%), 36 to 45 years old (*n* = 46, 15.5%), and the least above 46 years old (*n* = 29, 9.8%). A total of 209 (70.4%) worked in public kindergartens, and 88 (29.6%) in private ones.

Most teachers were in kindergarten year 2 (*n* = 130, 43.8%), followed by teachers in kindergarten year 3 (102, 34.3%), and the fewest in kindergarten year 1 (*n* = 65, 21.9%). The highest percentage by education level held a bachelor’s degree (*n* = 159, 53.5%), followed by a junior college degree (*n* = 115, 38.7%), master’s degree or above (*n* = 15, 5.1%), and secondary vocational school education (*n* = 8, 2.7%). By monthly income, the largest group was 3001–4000 (*n* = 105, 35.4%), followed by 4001–5000 (*n* = 77; 25.9%), 2001–3000 (*n* = 56, 18.9%), more than 5000 (*n* = 38, 12.8%), and less than 2000 (*n* = 21, 7.1%; Table 1).

### 3.2. Bias Test Results

First, to ensure non-response bias in the data of this study, a paired *t*-test was conducted on the demographic data of the initial and final 20 individuals in the questionnaire. The results showed compliance and no significant differences were found.

Second, to measure the common method bias for the data in this study, we used the methods of Podsakoff et al. [74] and Kock [75]. The rate of extraction of a single factor was 29.437%, which is below the threshold of 40%; the VIF values were all below the threshold of 3.3 [72]. This suggests that common method bias was not a serious problem in this study.

### 3.3. Measurement Model

CR, AVE, discriminant validity, and outer loading were used separately to ensure the quality of the model. The results of the analysis are as follows: Cronbach’s α and CR of the data in this study are greater than 0.7, which indicates that the data are internally consistent, and the AVE values of the variables in the data in this study are greater than 0.5, which indicates that the convergent validity of the data meets the requirements [71], as shown in Table 2.

In this study, both Fornell and Larcker’s Test and the Heterotrait-Monotrait ratio (HTMT) test were used to identify discriminant validity. The results show that the square root of each variable’s AVE is greater than its correlation with other variables [71], and HTMT values are below 0.85. This indicates that the data discriminant validity of this study met the requirements [71], as shown in Table 3.

### 3.4. Structural Model

The collinearity results of this study showed that the VIF of each variable was less than 3, indicating that the collinearity requirement of this study was met. Subsequently, to test the hypotheses of this study, a structural model was used. Table 4 presents the specific path coefficients and significance test results.

AC (β = 0.432, *p* < 0.001) and AR (β = 0.259, *p* < 0.001) had a significant positive influence on PN, supporting Hypotheses H1a and H1c. AC had a significant positive influence on AR (β = 0.235, *p* < 0.01), supporting Hypothesis H1b. PN had a significant positive influence on KSB (β = 0.555, *p* < 0.001), supporting Hypothesis H2; there was no significant influence of JTS on both AC (β = −0.019, *p* = 0.736) and AR (β = 0.106, *p* = 0.091), and Hypotheses H3a and H3b were not supported; there was a significant positive influence of ETS on AC (β = 0.341, *p* < 0.001), supporting Hypothesis H4a; there was no significant influence of ETS on AR (β = 0.027, *p* = 0.689), and Hypothesis H4b was not supported; and LTS had a significant positive influence on both AC (β = 0.301, *p* < 0.001) and AR (β = 0.246, *p* < 0.001), supporting Hypotheses H5a and H5b.

We used standardized root mean square residuals (SRMR) to judge the goodness of fit (GOF) of the model. The results show that the SRMR in this study is 0.052, which is less than the criterial value of 0.08, which indicates that the fit of this study meets the requirements [76].

### 3.5. Multigroup Analysis

Since preschool teachers’ knowledge sharing can take place through both interpersonal sharing and Internet sharing contexts, it is necessary to group the two contexts and compare how the path coefficients differ in different settings. A *p*-value (SS vs. SI) < 0.05 in Hensel’s MGA method indicates a significant difference between the path coefficients of the two data groups. Under different groups, AC had a significant influence on PN (*p* < 0.05), ETS had a significant influence on the AR (*p* < 0.05), and LTS had a significant influence on the AR (*p* < 0.05) (Table 5).

## 4. Discussion and Conclusions

### 4.1. Key Findings

This study investigated the influence of preschool teachers’ thinking styles on knowledge-sharing behaviors using a Norm Activation Model. The study found that the legislative thinking style has a significant positive influence on awareness of consequences and attribution of responsibility. Executive thinking style only had a significant positive influence on awareness of consequences and no significant influence on ascription of responsibility. Judicial thinking had no significant influence on either awareness of the consequences or ascription of responsibility. Awareness of consequences and ascription of responsibility had a significant positive influence on personal norms, whereas awareness of consequences had a significant positive influence on ascription of responsibility and personal norms had a significant positive influence on knowledge-sharing behaviors. We also explored the differences through two different sharing contexts: interpersonal sharing/Internet sharing.

First, legislative thinking has a significant positive influence on awareness of the consequences and ascription of responsibility. People with legislative thinking are more inclined to perform creative work [60], and creative teachers are good at using their judgments in practice [64]. This means that preschool teachers with legislative thinking may be more aware of the potential consequences of their ideas and actions, and they may be better equipped to anticipate and assess the consequences of not sharing their knowledge and have a greater awareness of consequences. When teachers use their own developed teaching methods, they create a sense of ownership and responsibility [64]. This means that teachers with a legislative thinking approach are more likely to consider responsibility for the consequences of not sharing knowledge. Therefore, preschool teachers with a legislative thinking approach are more likely to have stronger ascriptions of responsibility.

Second, executive thinking has a significant positive influence on awareness of consequences. Executive thinking people are more inclined to obey authority [63], consider the failure to follow the masses very dangerous [54], and are more susceptible to collectivist incentives to share knowledge [58]. That is, preschool teachers with an executive thinking style are more afraid of taking risks and have stronger awareness of the consequences of not sharing their knowledge. However, executive thinking did not have a significant influence on ascription of responsibility. A possible explanation is that preschool teachers with an executive thinking style are more inclined to obey arrangements, follow rules, and perform procedural tasks [47]; however, this does not mean that they have a sense of responsibility for the consequences of not sharing knowledge, and they may believe that if knowledge sharing is not a necessary task, they do not necessarily perform it.

Third, contrary to our prediction, judicial thinking did not have a significant influence on awareness of consequences and ascription of responsibility. A possible reason is that, although judicially thinking people are keen to evaluate, analyze, judge, and compare existing norms [57], they may not care about the consequences of their words and actions. That is, preschool teachers with a judicial thinking approach are not aware of the consequences of not sharing knowledge. Similarly, although people with a judicial thinking style can express their opinions, they usually do so in a critical manner, and the process of critical reflection does not imply responsibility for changes beyond their personal capacity [77]. Thus, being critical does not mean that one will be responsible, and preschool teachers with a judicial thinking approach do not have the sense of ascribing responsibility for the consequences of not sharing their knowledge.

Fourth, awareness of consequences and ascription of responsibility have a positive influence on personal norms, which supports existing research [36,37]. That is, the stronger the awareness of the consequences and ascription of responsibility, the more conducive it is to the establishment of personal norms. Therefore, preschool teachers tend to practice knowledge sharing as their moral obligation when they realize that it is beneficial to their own and their team’s professional development. Furthermore, when teachers consider knowledge sharing their responsibility as teachers, they fulfill their personal norms from the perspective of their role responsibilities. Awareness of consequences has a positive influence on the ascription of responsibility [41]. That is, the greater the awareness of the consequences, the greater the ascription of responsibility. This means that preschool teachers who are aware of the negative consequences of not participating in knowledge-sharing activities promote teacher responsibility.

Fifth, personal norms positively influence knowledge sharing. Personal norms are also known as ethical norms [39], and moral obligation can be a motivation to share knowledge [78,79]. People will share knowledge out of a sense of moral responsibility because they believe it is the right thing to do [44]. Thus, moral obligations positively influence preschool teachers’ knowledge-sharing behavior when they perceive it as pro-social behavior.

Finally, our subgroup analysis of different sharing contexts showed that the influence of executive thinking on ascription of responsibility, legislative thinking on ascription of responsibility, and awareness of consequences on personal norms were significantly different among preschool teachers who tended to engage in interpersonal sharing or Internet sharing. First, although preschool teachers who tended to engage in interpersonal sharing and Internet sharing of executive thinking did not have a significant influence on ascription of responsibility, there was a tendency for executive thinking styles in the interpersonal sharing group to positively influence ascription of responsibility. This may be because executive thinking preschool teachers are biased toward performing tasks, and less autonomous teachers tend to be followers [16]. When teachers with an executive style of thinking realize that knowledge sharing helps them in their work and is appreciated by colleagues and leaders [16,80], they will follow the lead of other teachers in sharing knowledge with colleagues and have the opportunity to realize, with the approval of colleagues and leaders, that knowledge sharing is also within the teacher’s responsibility. However, Internet sharing is a free and open environment in which teachers are not easily aware of their responsibility to share their knowledge, and do not consider it their responsibility to share with strangers.

Second, we found a significant influence of legislative thinking in preschool teachers on ascription of responsibility in the Internet sharing context, but no significant influence in the interpersonal sharing context. This may be because teachers have more autonomy when it comes to sharing on the Internet [14]. However, when sharing on the Internet, one needs to be self-disciplined and socially responsible and publish valuable information [40]. Therefore, when legislatively thinking preschool teachers share their knowledge on the Internet, they are more careful than when sharing it on campus and try to ensure that what they share is valuable and ascribe this process as their responsibility to do so.

Third, the influence of preschool teachers’ awareness of consequences on personal norms was more predictive in the Internet sharing context than in the interpersonal sharing scenario. A possible reason is that, although altruism can encourage people to share knowledge on the Internet [58], people will fear criticism on the Internet and therefore care more about their suggestions and the responses they receive, which will facilitate knowledge refinement [18]. That is, when sharing content on the Internet, preschool teachers will be aware of the consequences of sharing content that has no value. Therefore, sharing valuable knowledge can be seen as a personal norm.

### 4.2. Theoretical Contributions

This study contributes to the theoretical development of preschool teachers’ knowledge-sharing behaviors. First, although existing studies have explored the factors that influence teachers’ knowledge-sharing behaviors, most have focused on university teachers, and thus there is a lack of research on preschool teachers. This study adds reference material to preschool teachers’ knowledge-sharing behaviors.

Second, there is a lack of empirical research on the relationship between thinking styles and knowledge-sharing behaviors in studies of teachers’ knowledge-sharing behaviors. This study explores preschool teachers’ knowledge-sharing behavior from the perspective of three thinking styles (judicial, executive, and legislative), which not only expands our knowledge of the factors influencing knowledge-sharing behavior but also provides empirical evidence for the development of theory.

Again, this study applied the Norm Activation Model to preschool teachers to observe the factors that influence teachers’ knowledge sharing from a new perspective. The relationship between awareness of consequences, ascription of responsibility, personal norms, and knowledge-sharing behavior was confirmed, extending the use of the Norm Activation Model.

In addition, while previous studies have focused on teachers’ knowledge-sharing behaviors in a single context, this study enriches the literature on teachers’ knowledge-sharing behaviors in different contexts by comparing the differences between interpersonal sharing and internet sharing in groups.

Finally, this study may also have implications for research in other fields. Although this study was conducted with preschool teachers, it has some reference value for other fields, such as vocational education and training [81].

### 4.3. Practical Contributions

This study makes a practical contribution to promoting knowledge sharing among preschool teachers. The recommendations of this study are as follows:

First, schools should develop teachers’ awareness of consequences. This can be done by conducting knowledge-sharing seminars and training teachers to help them realize the importance of knowledge sharing. Teachers promote awareness of the consequences of knowledge-sharing behaviors by understanding the important role of knowledge sharing for the other faculty and their own development, as well as the negative effects of not sharing knowledge among teachers.

Second, we clarified the ascription of responsibility for teachers’ knowledge-sharing behavior. Schools should encourage teachers to share their insights with others [9] and include knowledge sharing among teachers in their work routines. Schools can promote mutual understanding among teachers through group learning, encourage teachers to help each other and learn together, deepen the sense of sharing among teachers, and carry a sense of sharing through teaching and life, so that the act of sharing knowledge among teachers becomes a habit and an integral part of teachers’ daily work.

Third, it is important to develop teachers’ legislative thinking. When facing difficult work problems, teachers are encouraged to prioritize using their own ways to solve problems, find new ideas and methods in the process of teaching and research, and cultivate their sense of innovation. Simultaneously, teachers are encouraged to collaborate and share new ideas with each other, creating opportunities for knowledge-sharing behaviors among teachers.

### 4.4. Limitations and Future Directions

The limitations of this study are as follows: First, the sample size was small, and the representativeness of the data may be problematic. Second, only Taiyuan, China, was selected for sampling in this study, and the results may have been influenced by other factors, leading to different results. It would thus be beneficial to encourage future research to draw samples from other Chinese cities as well as Western countries. Third, this study recruited preschool teachers through a local chat group, which may be underrepresentative, and future studies are encouraged to use other more representative research methods, such as big data analysis. Fourth, this study empirically examined only the three thinking styles defined by Sternberg [45] as judicial, executive, and legislative. However, there are multiple definitions for the classification of thinking styles, and we might have missed other thinking styles. Future research should encourage the exploration of other thinking styles.

## Figures and Tables

**Table 1:** Survey respondent characteristics.

Measures	Items	Frequency	Percent
Gender	Female	274	92.3%
	Male	23	7.7%
Age	<25	64	21.5%
	26~35	158	53.2%
	36~45	46	15.5%
	>45	29	9.8%
Kindergarten properties	public kindergartens	209	70.4%
	private kindergartens	88	29.6%
Kindergarten year	kindergarten year 1	65	21.9%
	kindergarten year 2	130	43.8%
	kindergarten year 3	102	34.3%
Education	secondary vocational school education	8	2.7%
	junior college degree	115	38.7%
	bachelor’s degree	159	53.5%
	master’s degree or above	15	5.1%
Income (CNY)	<2000	21	7.1%
	2001~3000	56	18.9%
	3001~4000	105	35.4%
	4001~5000	77	25.9%
	>5000	38	12.8%

**Table 2:** Reliability and Validity of Constructs.

Latent Variable	Item	Loading	Mean (SD)	Cronbach’s α	CR	AVE	R^2^
JTS	JTS1	0.818	3.476 (0.932)	0.853	0.906	0.763	-
	JTS2	0.904					
	JTS3	0.897					
ETS	ETS1	0.778	3.667 (0.772)	0.718	0.838	0.634	-
	ETS2	0.878					
	ETS3	0.724					
LTS	LTS1	0.840	3.697 (0.830)	0.884	0.919	0.740	-
	LTS2	0.846					
	LTS3	0.903					
	LTS4	0.851					
AC	AC1	0.833	4.099 (0.686)	0.859	0.904	0.703	0.232
	AC2	0.855					
	AC3	0.845					
	AC4	0.819					
AR	AR1	0.875	3.626 (0.826)	0.861	0.905	0.705	0.201
	AR2	0.815					
	AR3	0.835					
	AR4	0.832					
PN	PN1	0.854	3.953 (0.641)	0.766	0.865	0.682	0.330
	PN2	0.823					
	PN3	0.800					
KSB	KSB1	0.777	3.945 (0.663)	0.833	0.888	0.666	0.315
	KSB2	0.824					
	KSB3	0.816					
	KSB4	0.846					

AC—Awareness of consequences; AR—Ascription of responsibility; PN—Personal norm; JTS—Judicial thinking style; ETS—Executive thinking style; LTS—Legislative thinking style; KSB—Knowledge-sharing behavior.

**Table 3:** Discriminant Validity.

**Fornell–Larcker Criterion**							
	JTS	ETS	LTS	AC	AR	PN	KSB
JTS	0.874						
ETS	0.123	0.796					
LTS	0.378	0.149	0.860				
AC	0.137	0.384	0.345	0.838			
AR	0.234	0.167	0.371	0.345	0.840		
PN	0.259	0.285	0.332	0.521	0.408	0.826	
KSB	0.213	0.243	0.318	0.386	0.311	0.561	0.816
**Heterotrait-Monotrait Ratio**							
	JTS	ETS	LTS	AC	AR	PN	KSB
JTS							
ETS	0.151						
LTS	0.435	0.163					
AC	0.148	0.461	0.390				
AR	0.251	0.215	0.417	0.394			
PN	0.297	0.369	0.400	0.641	0.498		
KSB	0.248	0.307	0.366	0.454	0.364	0.694	

AC—Awareness of consequences; AR—Ascription of responsibility; PN—Personal norm; JTS—Judicial thinking style; ETS—Executive thinking style; LTS—Legislative thinking style; KSB—Knowledge-sharing behavior.

**Table 4:** Assessment of the Structural Model.

Hypothesis	β	STDEV	T-Statistic	*p*-Value	Result
H1a: AC -> PN	0.432	0.071	6.074	0.000	Support
H1b: AC -> AR	0.235	0.078	3.007	0.003	Support
H1c: AR -> PN	0.259	0.062	4.204	0.000	Support
H2: PN -> KSB	0.555	0.056	9.918	0.000	Support
H3a: JTS-> AC	−0.019	0.056	0.337	0.736	Reject
H3b: JTS -> AR	0.106	0.063	1.692	0.091	Reject
H4a: ETS -> AC	0.341	0.058	5.862	0.000	Support
H4b: ETS -> AR	0.027	0.069	0.400	0.689	Reject
H5a: LTS -> AC	0.301	0.060	5.005	0.000	Support
H5b: LTS -> AR	0.246	0.069	3.571	0.000	Support
Gender -> KSB	−0.097	0.193	0.504	0.614	-
Age -> KSB	0.056	0.055	1.016	0.310	-
Kindergarten year -> KSB	0.041	0.055	0.747	0.455	-
Edu -> KSB	−0.037	0.055	0.680	0.497	-
Income -> KSB	0.064	0.055	1.156	0.248	-
Kindergarten properties -> KSB	0.081	0.115	0.705	0.481	-

AC—Awareness of consequences; AR—Ascription of responsibility; PN—Personal norm; JTS—Judicial thinking style; ETS—Executive thinking style; LTS—Legislative thinking style; KSB—Knowledge-sharing behavior.

**Table 5:** Multigroup analysis results.

Hypothesis H6	β (XN) *n* = 212	β (XW) *n* = 85	*p* Value (SS)	*p* Value (SI)	*p* Value (SS vs. SI)	Result
AC -> PN	0.373	0.645	0.000	0.000	0.022	Support
AC -> AR	0.207	0.285	0.029	0.014	0.590	Reject
AR -> PN	0.267	0.171	0.001	0.016	0.358	Reject
PN -> KSB	0.555	0.544	0.000	0.000	0.944	Reject
JTS -> AC	−0.020	0.006	0.775	0.956	0.823	Reject
JTS -> AR	0.134	−0.010	0.097	0.929	0.292	Reject
ETS -> AC	0.367	0.312	0.000	0.001	0.632	Reject
ETS -> AR	0.107	−0.166	0.241	0.089	0.047	Support
LTS -> AC	0.247	0.425	0.000	0.000	0.124	Reject
LTS -> AR	0.145	0.502	0.061	0.000	0.022	Support

AC—Awareness of consequences; AR—Ascription of responsibility; PN—Personal norm; JTS—Judicial thinking style; ETS—Executive thinking style; LTS—Legislative thinking style; KSB—Knowledge-sharing behavior; SS—Sharing with colleagues at their school; SI—Sharing with the public through the Internet.

## Data Availability

The data presented in this study are available on request from the corresponding author. The data are not publicly available due to ethical reasons.

## Appendix A

**Table A1:** Scale.

Factor	Serial Num	Item	Reference
Judicial Thinking Style (JTS)	JTS 1	I like situations where I can compare and rate different ways of doing things.	Groza, Locander, and Howlett [47]
	JTS 2	I like to check and rate opposing points of view or conflicting ideas.	
	JTS 3	I like projects where I can study and rate different views or ideas.	
Executive Thinking Style (ETS)	ETS 1	I like to figure out how to solve a problem following certain (definite) rules.	
	ETS 2	I like projects that have a clear structure and set plan and goal.	
	ETS 3	I like to follow definite rules or directions when solving a problem or doing a task.	
Legislative Thinking Style (LTS)	LTS 1	When faced with a problem, I use my own ideas and strategies (ways) to solve it.	
	LTS 2	I like to play with my ideas and see how far they go.	
	LTS 3	I like problems where I can try my own ways of solving them.	
	LTS 4	When working on a task, I like to start with my own ideas.	
Awareness of Consequences (AC)	AC 1	Sharing can promote effective use of digital resources by teachers.	Kim, Woo, and Nam [39], Wang, Wang, Zhao, and Yang [41]
	AC 2	Sharing digital resources can inspire a community consciousness among teachers.	
	AC 3	Sharing digital resources facilitates teacher professional development.	
	AC 4	Sharing digital resources facilitates teachers’ knowledge innovation.	
Ascription of Responsibility (AR)	AR 1	I am co-responsible for the negative consequences of not sharing digital resources.	
	AR 2	I have a shared responsibility for sharing digital resources.	
	AR 3	I feel partially responsible for the negative consequences of teachers competing with each other.	
	AR 4	I feel partially responsible for the waste of digital resources.	
Personal Norm (PN)	PN 1	My personal values encourage me to share digital resources.	Zhang, Wang, and Zhou [67], Shin et al. [82]
	PN 2	My code of ethics encourages me to share digital resources.	
	PN 3	I have a moral obligation to share digital resources.	
Knowledge-sharing behavior (KSB)	KSB 1	Whenever others need digital resources, I always tell everything I know, without any hoarding.	Xue, Bradley, and Liang [68], Lu, Leung, and Koch [69]
	KSB 2	When I newly found a useful digital resource, I started to promote it and let more people learn about it.	
	KSB 3	I share digital resources with as many people as possible.	
	KSB 4	I usually actively share my digital resources.	
